# Meta-Analysis of Assessment of Total Oxidative Stress and Total Antioxidant Capacity in Patients with Periodontitis

**DOI:** 10.1155/2023/9949047

**Published:** 2023-10-30

**Authors:** Khadijah Mohideen, Krithika Chandrasekaran, Harsha Veeraraghavan, Shahul Hameed Faizee, Safal Dhungel, Snehashish Ghosh

**Affiliations:** ^1^Department of Oral Pathology and Microbiology, Sathyabama Dental College and Hospital, Sathyabama Institute of Science and Technology, Chennai 600119, India; ^2^Meenakshi Academy of Higher Education and Research, West K.K. Nagar, Chennai 600078, India; ^3^Sathyabama Dental College and Hospital, Sathyabama Institute of Science and Technology, Chennai 600119, India; ^4^Department of Orthodontics and Dentofacial Orthopaedics, Sathyabama Dental College and Hospital, Sathyabama Institute of Science and Technology, Chennai 600119, India; ^5^Department of Oral and Maxillofacial Surgery, College of Medical Sciences, Bharatpur, Nepal; ^6^Department of Oral Pathology, College of Medical Sciences, Bharatpur, Nepal

## Abstract

**Background:**

Periodontitis is intricately linked to oxidative stress-antioxidant (redox) imbalance. The antioxidant system scavenges the oxygen free radicals in biological fluids in patients with periodontitis. However, little is still known about the free radicals mediated oxidative stress and reductive ability of the antioxidant system. Thus, the present meta-analysis aims to quantitatively review the literature that assessed the oxidative stress marker total oxidative stress (TOS) and total antioxidant capacity (TAC) in various biological fluids of patients with periodontitis. *Methodology*. Electronic databases were searched for studies that assessed TOS and TAC levels in various biological samples of patients with periodontitis.

**Results:**

From the 1,812 articles identified, 1,754 were excluded based on title and abstract screening due to irrelevance to the topic of interest. A full-text assessment of the remaining 58 articles led to the selection of 42 articles that satisfied the inclusion criteria. Of these, only 24 studies had consistent data for quantitative analysis. The periodontitis group displayed significantly elevated TOS levels (*p* < 0.05) in serum, gingival crevicular fluid (GCF), and saliva samples in the studies evaluated. In contrast, the periodontitis group exhibited significantly attenuated TAC levels (*p* < 0.01) compared to healthy controls in plasma, serum, and GCF samples of the studies evaluated, which was insignificant in salivary samples (*p*=0.433). At the same time, the periodontitis group displayed insignificantly elevated TAC levels after periodontal therapy (*p*=0.130).

**Conclusions:**

The present meta-analysis showed significantly higher TOS and lower TAC in periodontitis, reflecting the elevated oxidative stress level than the control group. *Clinical Relevance*. Scientific rationale for the study: The imbalance between oxidants and antioxidants (oxidative stress (OS)) plays a critical role in the onset and progression of periodontitis; the assessment of the relationship between OS-related biomarkers in regional samples and systemic samples of patients with periodontitis helps us to evaluate the periodontal disease progression. The OS biomarker levels can be used to assess periodontal disease and therapeutic efficacy.

## 1. Introduction

Reactive oxygen species or free radicals are anions with one unpaired electron in the outer shell. The complex effect of enzymatic and nonenzymatic antioxidants scavenge or neutralize the free radical activity [1]. Indeed, when the antioxidant reserve system is exhausted by the excessive accumulation of free radicals, there is no scavenging of reactive oxygen species (ROS) nor their neutralization. The resultant disruption of the antioxidant barrier is directly responsible for oxidative stress (OS)/nitrosative stress-mediated modifications of biological components [2]. OS plays a primary role in the etiopathogenesis of many systemic diseases, such as diabetes and cardiovascular diseases, as well as a possible risk factor for chronic renal failure, rheumatoid arthritis, and neurodegenerative diseases [3]. Chronic inflammatory disease, like periodontitis, is also intricately associated with oxidative-reductive imbalance. Many studies have shown that OS is directly responsible for the progressive degradation of extracellular matrix components of the periodontal attachment apparatus [4]. Recent literature indicates that redox disturbances are intensified in periodontitis patients with comorbidities. Therefore, the present meta-analysis aims to assess the literature that evaluated the total oxidative and antioxidative capacity of various biological fluids in patients with periodontitis.

## 2. Materials and Methods

### 2.1. Registration of the Protocol

PRISMA guidelines have been strictly followed for study selection. The meta-analysis protocol was recorded in the PROSPERO database (CRD42021281819).

### 2.2. Question of Observation

Is there any significant difference in the total oxidative stress (TOS) and total antioxidant capacity (TAC) levels of biological fluid samples between patients with periodontitis and the healthy control group?

Based on the research question, the following components were formulated:

- Patient population: patients with periodontitis
- Exposure or marker of evaluation: mean and standard deviation value of TOS and TAC values
- Comparison: between patients with periodontitis and healthy control group
- Outcome: assessment of TOS and TAC in various biological fluid samples of patients with Periodontitis
- Study: collect the literature of cross-sectional and case-controlled studies that evaluated the status of TOS and TAC in Periodontitis and control from 2000 to 2023.

### 2.3. Literature Search

Electronic databases, including PubMed, ScienceDirect, Cochrane, Wiley Online Library, and Cross Reference, were searched for published articles addressing the TOS and TAC in patients with periodontitis and the control group between 2000 and 2023. The keywords were employed: “periodontal disease,” “total oxidative stress,” “oxidative stress,” and “total antioxidant capacity.”

### 2.4. Screening for Selection

Articles discussing oxidative stress and TAC in periodontitis were collected and screened for relevance based on the titles and abstracts.

### 2.5. Inclusion Criteria

Studies discussed the TOS and TAC in patients with periodontitis and the healthy control group;

- Both the case and control groups consisted of individuals who were in good systemic health, refraining from the use of antibiotics, anti-inflammatories, or any other medications. Moreover, they had not undergone any periodontal treatments within the past 3 months.
- The inclusion of studies examining systemic diseases in relation to periodontitis was contingent on the presence of two distinct evaluation groups: one for systemically healthy controls and another for the periodontitis-afflicted group.
- Studies investigating the correlation between periodontitis and smoking were considered only if they incorporated nonsmokers in both the periodontitis group and the control group as separate evaluation groups.
- Studies examining the impact of therapy on periodontitis were included only when they provided baseline values for both the periodontitis group and the control group.

Studies involving various biological fluid samples and expressed the assessed TOS and TAC values in mean, standard deviation, along with probability value:

- Papers provided consistent data to allow comparison of patient and control groups with other relevant studies.

### 2.6. Exclusion Criteria

Articles with the unmatched title and objectives:

- Studies that were duplicates, when they involved the same subjects and by the same authors.
- Observational studies exclusively focusing on pregnant women or children.
- The studies examined the therapeutic effect but did not include an evaluation of the control group.
- Studies included patients with systemic diseases or smokers but did not incorporate separate evaluation groups for systemically healthy individuals or nonsmokers in the periodontitis and control groups.
- Being literature, critical, or systematic reviews.
- Studies used other markers of oxidative stress and antioxidant capacity for evaluation.
- The articles provided incompatible data for the comparison between control and periodontitis groups with other studies.
- The studies expressed the results in Graphical representation without the accurate value display.
- Studies discussed the TOS and TAC values in other oral chronic inflammatory diseases.

### 2.7. Full-Text Retrieval and Evaluation

Two authors screened the titles and objectives of the collected studies and excluded the presentations at higher risk of bias from the quantitative synthesis based on predefined criteria. Two authors have independently evaluated the full text of each included study. Two authors have collected data for the factors considered in the meta-analysis. After considering all the particulars, the authors have selected the articles for eligibility criteria. The authors resolved disagreements by consensus. Finally, all the authors participated in manuscript preparation.

### 2.8. Data Segregation

The extracted information from the full text of selected articles as the author, year of publication, sample size, TOS and TAC measurements in Periodontitis, and control group expressed as the mean with standard deviation along with specific measurement units and assessment methods.

### 2.9. Statistical Analysis

The meta-analysis was performed by deriving the forest plot using the standard mean difference method using comprehensive meta-analysis software version 3 (Biostat Inc. Englewood, NJ, USA). The standardized mean difference values of TOS and TAC in periodontitis were evaluated at a 95% confidence interval (CI). Due to significant heterogeneity, a random-effects model was chosen for the analysis. The studies that expressed the TOS and TAC levels in similar units in each sample were only selected for the meta-analysis.

## 3. Results

**Figure 1:**
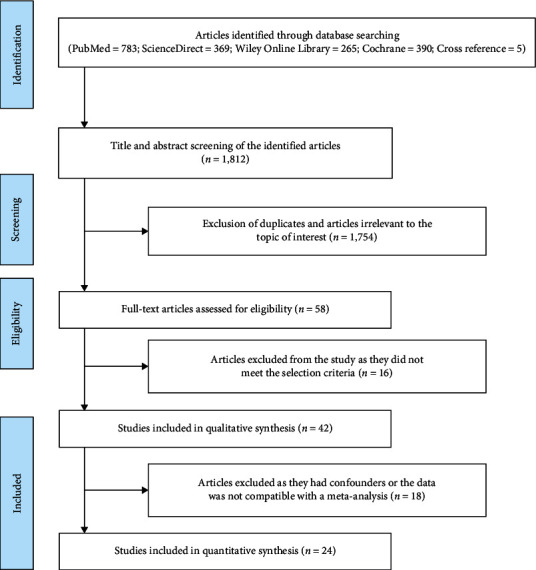
Flowchart for study selection.

PubMed search yielded 783 papers, ScienceDirect search yielded 369 papers, Wiley Online Library yielded 265 papers, Cochrane search yielded 390 papers, and Cross-reference search yielded five papers. After search refinement, 1,754 articles were excluded due to unmatched titles and abstracts, including four duplicated data reports and one animal study. After the extraction of these articles, 58 articles had titles relevant to the present work. The full text was retrieved for the screened articles. Papers not meeting the selection criteria (*n* = 16) were excluded. Finally, 42 articles with matched objectives were selected for the systematic review. Only 24 articles had data compatible with the meta-analysis (Figure 1).

Newcastle–Ottawa scale of included studies in the meta-analysis was displayed in Table 1 [5–46]. Collected TOS and TAC assessment data, criteria for periodontitis diagnosis, and other relevant findings from included articles in various biological fluid samples were tabulated in Tables 2 and 3, respectively [47–52]. The different methodologies utilized for assessing TOS and TAC values were also displayed in Tables 2 and 3 [53–61]. The pre and posttreatment mean values of TAC in different biological fluid samples in patients with periodontitis data from the included studies are presented in Table 4. The analysis of TOS levels after therapeutic intervention could not be performed due to the scarcity of published studies.

Selection—Case definition, case selection, control definition, and selection.

Comparability—Consideration of matching known and potential confounding factors.

Exposure—Securing patient records, interviewer blindness to groups, similarity ascertainment between groups, and nonresponse rate.

Each criterion was awarded with one star. The overall score was determined by summing the awarded stars. Studies receiving scores in the range of 6–9 were categorized as high-quality, those scoring between 3 and 5 were classified as fair-quality, and studies with scores ranging from 0 to 2 were deemed to be of poor quality. Importantly, it's worth noting that all the studies analyzed received scores exceeding 6, indicating a minimal risk of bias.

The periodontitis group displayed significantly elevated TOS levels (*p* < 0.05) in serum, gingival crevicular fluid (GCF), and saliva samples in the studies evaluated. In contrast, the periodontitis group exhibited significantly attenuated TAC levels (*p* < 0.01) compared to healthy controls in serum, plasma, and GCF samples of the studies evaluated, which was insignificant in salivary samples (*p*=0.433). At the same time, the periodontitis group displayed insignificantly elevated TAC levels after periodontal therapy (*p*=0.130).

**Figure 2:**
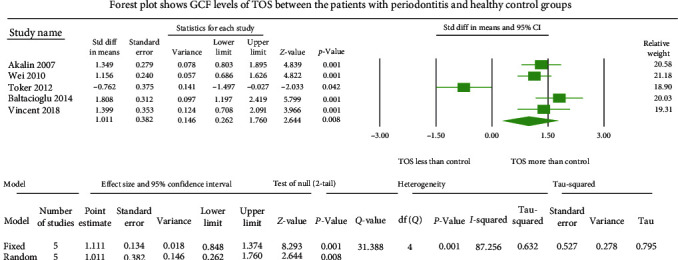
The forest plot shows standard mean difference estimates with 95% confidence intervals representing differences in GCF levels of TOS between the patients with periodontitis and healthy controls.

**Figure 3:**
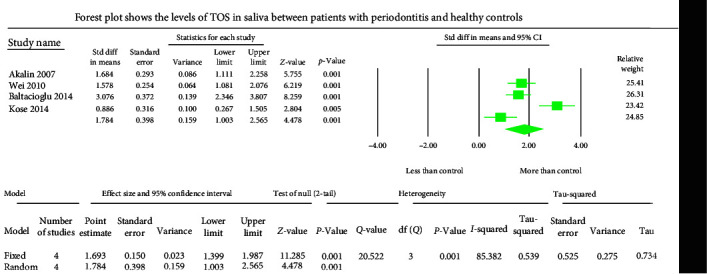
The forest plot depicts standard mean difference estimates with 95% confidence intervals representing differences in salivary levels of TOS between patients with periodontitis and healthy controls.

**Figure 4:**
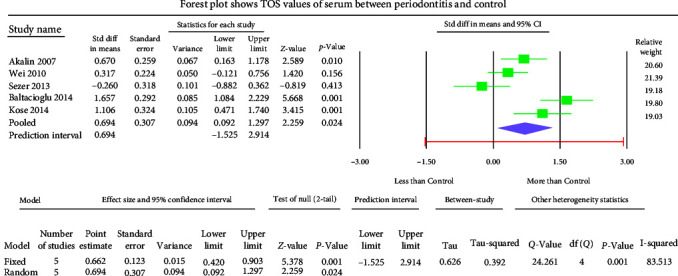
The forest plot displays standard mean difference estimates with 95% confidence intervals representing differences in serum levels of TOS between patients with periodontitis and healthy controls.

The GCF samples showed an overall standard mean difference TOS value of 1.011 *µ*mol H_2_O_2_ equivalent (Eq)/l with 95% CI (0.262–1.760) (Figure 2). The salivary samples displayed an overall standard mean difference TOS value of 1.784 *µ*mol H_2_O_2_ Eq/l with 95% CI (1.003–2.565) (Figure 3). The serum samples depicted an overall standard mean difference TOS value of 0.694 *µ*mol H_2_O_2_ Eq/l with 95% CI (0.092–1.297) (Figure 4).

**Figure 5:**
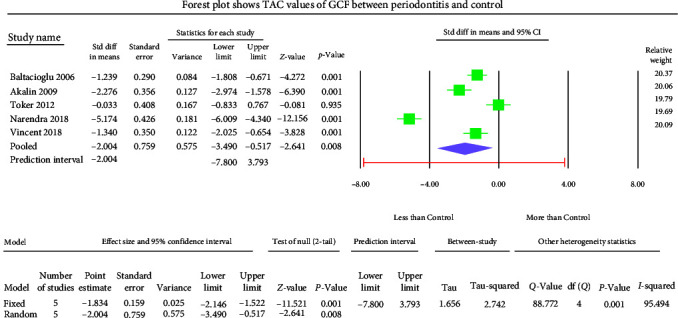
The forest plot shows standard mean difference values of TAC in GCF samples with 95% confidence intervals between patients with the periodontitis group and healthy controls.

**Figure 6:**
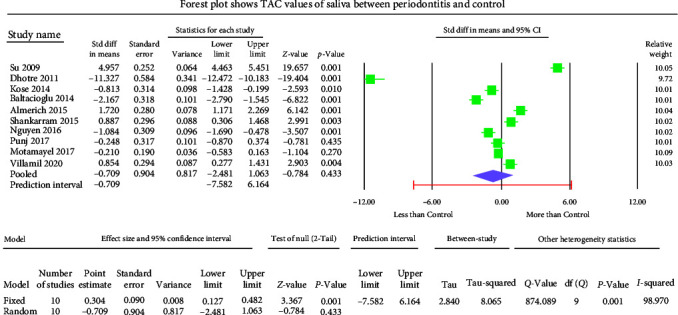
The forest plot shows salivary TAC standard mean difference values with 95% confidence intervals between patients with the periodontitis group and healthy controls.

**Figure 7:**
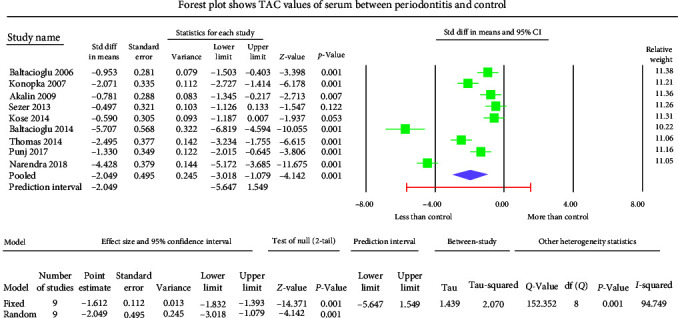
The forest plot shows serum TAC standard mean difference values with 95% confidence intervals between patients with periodontitis and healthy controls.

**Figure 8:**
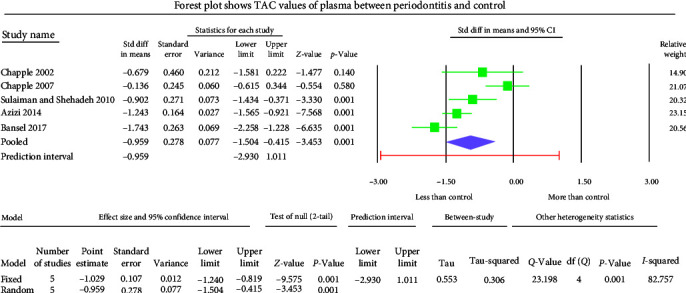
The forest plot shows plasma TAC standard mean difference values with 95% confidence intervals between patients with periodontitis and healthy controls.

**Figure 9:**
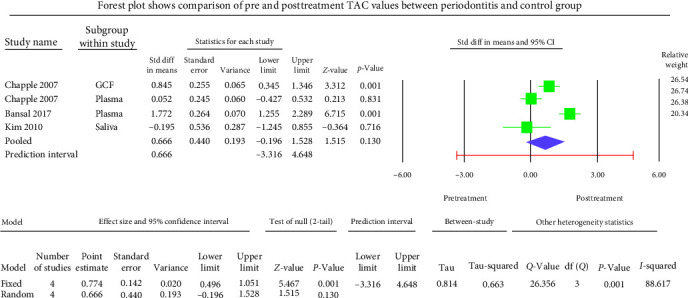
Meta-analysis of TAC levels between before and after treatment of periodontitis group.

The GCF samples showed an overall mean difference TAC value of −2.004 mmol Trolox equivalent (TEq)/l with 95% CI (−3.490 to −0.517) (Figure 5). The salivary samples displayed an overall standard mean difference TAC value of −0.709 mmol TEq/l with 95% CI (−2.481 to 1.063) (Figure 6). The serum samples showed an overall standard mean difference TAC value of −2.049 mmol TEq/l with 95% CI (−3.018 to −1.079) (Figure 7). The plasma samples showed an overall standard mean difference TAC value of −0.959 *µ*mol TEq/l with 95% CI (−1.504 to −0.415) (Figure 8). The overall standard mean difference TAC value pre and posttherapy is 0.666 *µ*mol TEq/l with 95% CI (−0.196 to 1.528) (Figure 9).

The meta-analysis of the TOS assessment presented high heterogeneity, reflected by the *I*^2^ values 87.256, 85.382, and 83.513 in Figure 2–4, respectively. The meta-analysis of the TAC assessment presented high heterogeneity, reflected by the *I*^2^ values 95.494, 98.970, 94.749, 82.757, and 88.617 in Figure 5, Figure 6, Figure 7, Figure 8, and Figure 9, respectively. The different methodologies utilized to measure TOS and TAC values could cause high heterogeneity.

### 3.1. Publication Bias

Studies included in this assessment of TOS in periodontitis meta-analysis showed Egger's regression intercept values 9.162, −6.856, 3.689 with two-tailed *p*-values 0.45, 0.45, and 0.701 in saliva, GCF, and serum samples, respectively, indicating a low risk of publication bias of included studies in meta-analysis. Studies included in this assessment of TAC in periodontitis meta-analysis showed Egger's regression intercept values −12.79, 1.895, −23.06 with two-tailed *p*-values 0.48, 0.67, and 0.07 in GCF, plasma, and saliva samples, respectively, denoting a lower risk of publication bias of included studies in meta-analysis. Studies included in this assessment of serum TAC in periodontitis meta-analysis showed Kendall's *S* statistic (*P*–*Q*) value of −20.0 with two-tailed *p*-values of 0.051 (Begg and Mazumdar's test for rank correlation), denoting a moderate risk of publication bias of included studies in the salivary assessment of TAC meta-analysis. The studies included the posttherapy TAC assessment in periodontitis meta-analysis showed Eggers regression intercept value of −2.67 with a two-tailed *p*-value of 0.73, indicating a low risk of publication bias of included studies in meta-analysis.

## 4. Discussion

Despite the increasing knowledge of the etiopathogenesis of inflammatory periodontal diseases, there are no definitive indicators for objectivizing the diagnosis, determining the disease's severity, and evaluating treatment results. Hence, the present meta-analysis assessed the literature evaluated the oxidative stress markers such as TOS and TAC levels in the blood, GCF, and both stimulated and nonstimulated saliva in patients with periodontitis to find the validity of these markers in determining the diagnosis and prognosis of periodontitis as well as the treatment effects. In the present meta-analysis, Out of 10 studies of TOS assessment in serum, GCF, and saliva involving 260 patients in the study group and 212 in the control group, seven studies proved a significantly higher TOS in patients with periodontitis [9, 16, 19, 26–28, 42]. They also reported a strong correlation of TOS with the clinical parameters of periodontitis [12, 16]. This fact is unsurprising since the stimulated saliva secreted by the parotid gland is the primary source of free radicals (ROS) [62, 63]. However, Zhang et al. [64] showed no difference in the salivary TOS levels between periodontitis and healthy controls. Further, they found that a high bacterial load did not depict any correlation with salivary TOS values [64]. Toczewska et al. [65] found a weak correlation of TOS values with clinical periodontal parameters. These differences might be due to the selection criteria of patients in different studies. Since other factors related to subjects such as age, smoking, gender, and nutrition would yield distinct effects on the alteration of OS parameters; thus, it is essential to consider these factors when considering this parameter as a potential marker of periodontitis. Therefore, patient selection could be a crucial parameter influencing salivary levels of TOS.

The present meta-analysis of the evaluated studies displayed significantly elevated TOS levels (*p* < 0.05) in GCF, saliva, and serum samples of the periodontitis group with the overall standardized mean difference value of 1.011, 1.784, and 0.694 *µ*mol H_2_O_2_ Eq/l, respectively.

Out of 39 studies of TAC assessment in plasma, serum, GCF, and saliva, involving 1,418 patients in the study group and 1,340 in the control group, except 11 studies, the remaining 31 studies proved a significant decrease in the TAC in patients with periodontitis when compared to the clinically healthy periodontium [5, 6, 8, 14, 21–24, 27, 38, 39]. Chapple et al. [8] reported that GCF/salivary TAC values were significantly higher in patients with periodontitis than in the control group. The authors attributed that the initial increase in antioxidant response in periodontitis is due to local reactive or adaptive response to a first phase increase of oxidative burst (OS) occurring with periodontal inflammation. The adaptive antioxidant defense might decrease over time as ROS production becomes chronic. The excess utilization of antioxidants to neutralize the exaggerated ROS activity during periodontal inflammation results in the depletion of TAC levels in participants with periodontitis. The facts mentioned above might account for the different results reported in the various studies evaluating TAC levels in patients with periodontitis. Wei et al. [16] and Baltacıoğlu et al. [26] found significant correlations between salivary/serum TOS levels and clinical parameters of periodontitis (plaque index, gingival index, probing depth, and clinical attachment level). Baser et al. [33] and Zhang et al. [64] studies depicted plasma and salivary TAC values correlated with clinical periodontal parameters. Zhang et al. [64] further found that a high bacterial load did not exhibit any correlation with salivary TAC levels. Their study carried out a multifactorial analysis, and they depicted that out of many factors, the diagnosis of periodontitis was significantly related to TAC salivary values only regardless of other variables such as age, gender, smoking habits, or presence of periodontal pathogens in saliva. They also found a significant inverse relationship between salivary TAC and the clinical attachment level of periodontitis [64]. Toczewska et al. [65] research found a significant reduction of GCF total antioxidant activity in periodontal pockets compared to other gingival regions, and the extent of this reduction did not correlate with the different stages of periodontitis and weakly correlated with clinical periodontal parameters. Becerik et al. [66] stated that a significant decrease in the GCF antioxidant capacity (ferric reducing antioxidant power) in patients with periodontitis also displayed an inverse correlation with the clinical parameters of periodontitis, such as the clinical attachment level and pocket depth. Some previous studies demonstrated that reduced salivary TAC values correlated with increased inflammatory burden in periodontitis [11, 67, 68]. The three included studies of the present meta-analysis showed that periodontal therapy significantly improved salivary and serum TAC values in patients with periodontitis. It is also suggested that shifting the salivary/GCF redox balance in favor of the oxidative reactions (↓TAC, ↑TOS) predisposes to oxidative damage to proteins, lipids, and DNA in the periodontal tissue, which leads to progressive degradation of the periodontal attachment apparatus [4, 69]. Su et al. [13] and Panjamurthy et al. [70] reported higher serum, GCF, and salivary TAC values in periodontitis participants. However, some studies also showed that salivary TAC increases or remains at the same level in periodontitis patients compared to healthy controls [6, 32, 71]. Another study reported that neither gingivitis nor smoking habits influence salivary TAC values [72]. The variations in the results of TAC between different studies could be due to different analytical methods utilized in assessing TAC values.

The present meta-analysis of the evaluated studies exhibited significantly attenuated TAC levels (*p* < 0.01) in the periodontitis group compared to healthy controls in GCF, serum, and plasma samples, which was insignificant in salivary samples (*p*=0.433). The overall mean difference of TAC value in GCF, salivary, serum, and plasma samples were −2.004, −0.709, −2.049, and −0.959 *µ*mol TEq/l.

At the same time, the periodontitis group displayed insignificantly (*p*=0.130) elevated TAC levels after periodontal therapy. The overall standard mean difference TAC value upon comparison of pre and posttherapy was 0.666 *µ*mol TEq/l.

Although high heterogeneity was detected among included studies of the present meta-analysis, our results still indicate that periodontitis has statistically correlated with some local OS biomarkers, and oxidative stress played a critical role in the pathological process of periodontitis. Thus, TOS and TAC may be helpful and practical biomarkers for evaluating oxidative injury in periodontal tissues.

## 5. Conclusions

In conclusion, the present meta-analysis supports the rationale that there is a direct link between periodontitis and OS-related biomarkers in the local site. The imbalance of ROS and antioxidant systems may contribute to functional and structural remodeling that favors the occurrence of periodontitis. Furthermore, these two measurements can potentially evaluate the interaction between periodontal and systemic status and the effectiveness of periodontal treatment. Considering the above facts, it may be speculated that oxidative stress is an essential factor in periodontitis. Studying the antioxidant defense mechanisms may be regarded as a valuable biomarker that will help better understand the underlying pathology of tissue damage and novel therapeutic interventional strategies. Therefore, it is necessary to conduct further research on a larger number of patients with periodontitis to understand the oxidative stress and antioxidant status imbalance reported in patients with periodontitis.

## Figures and Tables

**Table 1:** Newcastle–Ottawa Scale for studies included in the meta-analysis.

Study	Selection	Comparison	Exposure	Total scores
Chapple et al. [5]	^*∗*^ ^*∗*^ ^*∗*^ ^*∗*^	^*∗*^ ^*∗*^	^*∗*^ ^*∗*^	8
Brock et al. [6]	^*∗*^ ^*∗*^ ^*∗*^ ^*∗*^	^*∗*^ ^*∗*^	^*∗*^ ^*∗*^ ^*∗*^	9
Baltacıoğlu et al. [7]	^*∗*^ ^*∗*^ ^*∗*^ ^*∗*^	^*∗*^ ^*∗*^	^*∗*^ ^*∗*^	8
Chapple et al. [8]	^*∗*^ ^*∗*^ ^*∗*^ ^*∗*^	^*∗*^ ^*∗*^	^*∗*^ ^*∗*^ ^*∗*^	9
Akalın et al. [9]	^*∗*^ ^*∗*^ ^*∗*^ ^*∗*^	^*∗*^ ^*∗*^	^*∗*^ ^*∗*^	8
Konopka [10]	^*∗*^ ^*∗*^ ^*∗*^ ^*∗*^	^*∗*^ ^*∗*^	^*∗*^ ^*∗*^	8
Guentch et al. [11]	^*∗*^ ^*∗*^ ^*∗*^ ^*∗*^	^*∗*^ ^*∗*^	^*∗*^ ^*∗*^ ^*∗*^	9
Akalın et al. [12]	^*∗*^ ^*∗*^ ^*∗*^ ^*∗*^	^*∗*^ ^*∗*^	^*∗*^ ^*∗*^	8
Su et al. [13]	^*∗*^ ^*∗*^ ^*∗*^ ^*∗*^	^*∗*^ ^*∗*^	^*∗*^ ^*∗*^	8
Kim et al. [14]	^*∗*^ ^*∗*^ ^*∗*^ ^*∗*^	^*∗*^ ^*∗*^	^*∗*^ ^*∗*^ ^*∗*^	9
Sulaiman and Shehadeh [15]	^*∗*^ ^*∗*^ ^*∗*^ ^*∗*^	^*∗*^ ^*∗*^	^*∗*^ ^*∗*^ ^*∗*^	9
Wei et al. [16]	^*∗*^ ^*∗*^ ^*∗*^ ^*∗*^	^*∗*^ ^*∗*^	^*∗*^ ^*∗*^	8
Dhotre et al. [17]	^*∗*^ ^*∗*^ ^*∗*^ ^*∗*^	^*∗*^ ^*∗*^	^*∗*^ ^*∗*^	8
Dhotre et al. [18]	^*∗*^ ^*∗*^ ^*∗*^ ^*∗*^	^*∗*^ ^*∗*^	^*∗*^ ^*∗*^	8
Esen et al. [19]	^*∗*^ ^*∗*^ ^*∗*^ ^*∗*^	^*∗*^ ^*∗*^	^*∗*^ ^*∗*^	8
Konuganti et al. [20]	^*∗*^ ^*∗*^ ^*∗*^ ^*∗*^	^*∗*^ ^*∗*^	^*∗*^	7
Toker et al. [21]	^*∗*^ ^*∗*^ ^*∗*^ ^*∗*^	^*∗*^ ^*∗*^	^*∗*^ ^*∗*^	8
Akpinar et al. [22]	^*∗*^ ^*∗*^ ^*∗*^ ^*∗*^	^*∗*^ ^*∗*^	^*∗*^ ^*∗*^	8
Novakovic et al. [23]	^*∗*^ ^*∗*^ ^*∗*^ ^*∗*^	^*∗*^ ^*∗*^	^*∗*^ ^*∗*^	8
Sezer et al. [24]	^*∗*^ ^*∗*^ ^*∗*^ ^*∗*^	^*∗*^ ^*∗*^	^*∗*^ ^*∗*^	8
Azizi et al. [25]	^*∗*^ ^*∗*^ ^*∗*^ ^*∗*^	^*∗*^ ^*∗*^	^*∗*^ ^*∗*^	8
Baltacıoğlu et al. [26]	^*∗*^ ^*∗*^ ^*∗*^ ^*∗*^	^*∗*^ ^*∗*^	^*∗*^ ^*∗*^	8
Bostanci et al. [27]	^*∗*^ ^*∗*^ ^*∗*^ ^*∗*^	^*∗*^ ^*∗*^	^*∗*^ ^*∗*^ ^*∗*^	9
Kose et al. [28]	^*∗*^ ^*∗*^ ^*∗*^ ^*∗*^	^*∗*^ ^*∗*^	^*∗*^ ^*∗*^ ^*∗*^	9
Miricescu et al. [29]	^*∗*^ ^*∗*^ ^*∗*^ ^*∗*^	^*∗*^ ^*∗*^	^*∗*^ ^*∗*^	8
Shirzaiy et al. [30]	^*∗*^ ^*∗*^ ^*∗*^ ^*∗*^	^*∗*^ ^*∗*^	^*∗*^ ^*∗*^	8
Thomas et al. [31]	^*∗*^ ^*∗*^ ^*∗*^ ^*∗*^	^*∗*^ ^*∗*^	^*∗*^ ^*∗*^ ^*∗*^	9
Almerich-Silla et al. [32]	^*∗*^ ^*∗*^ ^*∗*^ ^*∗*^	^*∗*^	^*∗*^	6
Baser et al. [33]	^*∗*^ ^*∗*^ ^*∗*^ ^*∗*^	^*∗*^ ^*∗*^	^*∗*^ ^*∗*^	8
Shankarram et al. [34]	^*∗*^ ^*∗*^ ^*∗*^ ^*∗*^	^*∗*^ ^*∗*^	^*∗*^	7
Nguyen et al. [35]	^*∗*^ ^*∗*^ ^*∗*^ ^*∗*^	^*∗*^ ^*∗*^	^*∗*^ ^*∗*^	8
Atabay et al. [36]	^*∗*^ ^*∗*^ ^*∗*^ ^*∗*^	^*∗*^ ^*∗*^	^*∗*^ ^*∗*^ ^*∗*^	9
Bansel et al. [37]	^*∗*^ ^*∗*^ ^*∗*^ ^*∗*^	^*∗*^ ^*∗*^	^*∗*^ ^*∗*^	8
Ahmadi-Motamayel et al. [38]	^*∗*^ ^*∗*^ ^*∗*^ ^*∗*^	^*∗*^ ^*∗*^	^*∗*^ ^*∗*^	8
Punj et al. [39]	^*∗*^ ^*∗*^ ^*∗*^ ^*∗*^	^*∗*^ ^*∗*^	^*∗*^ ^*∗*^	8
Narendra et al. [40]	^*∗*^ ^*∗*^ ^*∗*^ ^*∗*^	^*∗*^ ^*∗*^	^*∗*^ ^*∗*^	8
Verma et al. [41]	^*∗*^ ^*∗*^ ^*∗*^ ^*∗*^	^*∗*^ ^*∗*^	^*∗*^ ^*∗*^	8
Vincent et al. [42]	^*∗*^ ^*∗*^ ^*∗*^ ^*∗*^	^*∗*^ ^*∗*^	^*∗*^ ^*∗*^	8
Sánchez-Villamil et al. [43]	^*∗*^ ^*∗*^ ^*∗*^ ^*∗*^	^*∗*^ ^*∗*^	^*∗*^ ^*∗*^	8
Senouci et al. [44]	^*∗*^ ^*∗*^ ^*∗*^ ^*∗*^	^*∗*^ ^*∗*^	^*∗*^ ^*∗*^	8
Thomas et al. [45]	^*∗*^ ^*∗*^ ^*∗*^ ^*∗*^	^*∗*^	^*∗*^	6
Verghese et al. [46]	^*∗*^ ^*∗*^ ^*∗*^ ^*∗*^	^*∗*^ ^*∗*^	^*∗*^ ^*∗*^ ^*∗*^	9

**Table 2:** The mean values of TOS in various biological fluid samples between healthy controls and patients with periodontitis in the included studies of quantitative synthesis.

Study name	Country	Study design	Criteria	Age years case/control mean ± SD or range	Sample type	Unit of measurement	Periodontitis		Control		*p*-Value
							Mean ± SD or median (upper–lower value)	Sample size (M/F)	Mean ± SD median (upper– lower value)	Sample size (M/F)	
Akalın et al. [9]	Turkey	CC	Armitage [47]	40.66 ± 5.31/38.5 ± 6.10	Serum	*µ*mol	22.5 ± 17.21	36 (19/17)	13.77 ± 2.381	28 (13/15)	<0.05
					Saliva	*µ*mol	6.03 ± 1.37		4.16 ± 0.63		<0.05
					GCF	*µ*mol	39.2 ± 5.95		31.4 ± 5.54		<0.05
Wei et al. [16]	China	PS	Armitage [47]	40.1 ± 7.3/42.1 ± 7.7	Serum	mmol	24.8 ± 18.53	48 (27/21)	19.21 ± 16.246	35 (19/16)	<0.05
					Saliva	mmol	9.12 ± 1.77		6.75 ± 1.02		<0.05
					GCF	mmol	50.9 ± 6.33		42.76 ± 7.94		<0.05
Esen et al. [19]	Turkey	CS	AL ≥ 4 mm and PPD ≥ 5 mm	42.85 ± 9.6/40.05 ± 9.8	Serum	*µ*mol H_2_O_2_ equiv./l	7.115 (4.920– 8.055)	20 (4/16)	6.935 (5.655– 8.755)	20 (4/16)	0.920
					GCF		0.285 (0.070–0.360)		0.010 (0.010– 0.415)		0.030
Toker et al. [21]	Turkey	Interventional study	CAL > 30% of sites; PPD ≥ 5 mm	38.7 ± 5.9/38.0 ± 7.2	GCF	*µ*mol H_2_O_2_ Eq/l	10.06 ± 0.22	15 (7/8)	10.4 ± 0.48	10 (6/4)	<0.05
Akpinar [22]	Turkey	Interventional	Armitage [47]	37.7 ± 5.9/37.0 ± 7.4	GCF	*µ*mol H_2_O_2_ equiv./l	(9.4–10.7) 10	15 (7/8)	(9.7–11.3) 10.6	10 (5/5)	
Sezer [24]	Turkey	CS	–	45.50 ± 7.50/40.75 ± 10.26	Serum	*µ*mol H_2_O_2_ Eq/l	19.48 ± 7.91	20 (6/14)	21.47 ± 7.39	20 (6/14)	
Baltacıoğlu et al. [26]	Turkey	CS and CC	Armitage [47]	32.55 ± 5.32/30.10 ± 4.06	Serum	*µ*mol	16.8 ± 2.467	33 (16/17)	13.77 ± 0.567	30 (16/14)	<0.05
					Saliva	*µ*mol	6.27 ± 0.844		4.167 ± 0.444		<0.05
					GCF	*µ*mol H_2_O_2_ Eq/l	27.5 ± 4.96	30	19.75 ± 3.46	28	<0.05
Bostanci [27]	Turkey	Interventional studies	Armitage [47]	38.80 ± 4.87/37.33 ± 5.67	GCF	*µ*mol H_2_O_2_ Eq/l	9.74 (8.75–12.25)	15 (6/9)	5.25(4.05–7.72)	15 (7/8)	<0.05
					Serum		4.2 (3.25–6.85)		3.86(2.24–5.25)		>0.05
Kose et al. [28]	Turkey	Comparative study	Armitage [47]	27–51 years	Serum	*µ*mol H_2_O_2_ Eq/l	16 ± 4.01	22 (11/11)	12.19 ± 2.77	22 (12/10)	<0.05
					Saliva		6.8 ± 3.49		4.41 ± 1.54		<0.05
Vincent et al. [42]	India	Comparative study	Armitage [48]	25–65 years	GCF	*µ*mol/l	9.08 ± 3.7	20 (10/10)	5.2 ± 1.3	20 (11/9)	<0.001

The method utilized for the assessment of TOS values in various biological samples was Erel [53]. SD, standard deviation; Cont, control; H_2_O_2_, hydrogen peroxide; Eq, equivalent; CC, case–control; PS, prospective study; CS, cross-sectional study.

**Table 3:** The mean values of TAC in various biological fluid samples between healthy controls and patients with periodontitis in the included studies included in the meta-analysis.

Author name and Year	Country	Study design	Criteria	Age years case/control mean ± SD or range	Sample type	Unit	Periodontitis		Control		*p*-Value	Method of assessment
							Mean ± SD or median (upper–lower value)	Sample size (M/F)	Mean ± SD or median (upper–lower value)	Sample size (M/F)		
Chapple et al. [5]	UK	CS	Gustafsson et al. [49]	46.1/46.9	Plasma	*µ*mol TEq/l	501.8 ± 123	10 (5/5)	577.9 ± 99.8	10 (5/5)	>0.05	Chapple et al. [54]
Brock et al. [6]	UK	CC	PPD ≥ 5 mm at least two sites per quadrant, BOP, radiographic LB ≥ 30%	43.5 (23–60) /44.7 (24–63)	Saliva	nmol/30s sample	0.18 ± 0.08	17 (7/10)	0.14 ± 0.06	17 (7/10)	NS	Chapple et al. [54]
					GCF	*µ*mol	0.14 ± 0.06		0.18 ± 0.08		<0.001	
Baltacıoğlu et al. [7]	Turkey	Comparative study	Armitage [47]	37.4 ± 5.4/37.1 ± 4.2	Serum	mM Trolox equivalent	0.53 ± 0.19	31 (F)	0.72 ± 0.21	26 (F)	<0.05	Erel [55]
					GCF		0.07 ± 0.03		0.12 ± 0.05			
					GCF TAOC/30 s		0.36 ± 0.12		0.50 ± 0.16			
Chapple et al. [8]	UK	Interventional	Brock et al. [6]	32–60	Plasma	*µ*mol TEq	507 ± 92	35 (12/23)	520 ± 100	32 (N/A)	0.57	Chapple et al. [54] Maxwell et al. [56]
					GCF		680 ± 371		1129 ± 722		<0.0001	
Konopka et al. [10]	Poland	CC	Lindhe et al. [50]	31.5/33.2	Venous Blood	mmol/l	1.68 ± 0.13	30 (15/15)	1.94 ± 0.12	25 (10/15)	<0.001	ABTS reduction method
					Gingival Blood		1.81 ± 0.18		1.94 ± 0.13		0.029	
Guentsch et al. [11]	Germany	Interventional study	At least 30% of teeth with PPD >5 mm	46.3 ± 13.1/34.1 ± 11.8	Saliva	*μ*mol/ml	0.37 ± 0.24	15 (6/9)	0.52 ± 0.20	15 (7/8)	<0.05	Popov and Lewin [57]
Akalin et al. [12]	Turkey	CS	Armitage [47]	29.3 ± 3.94/29.73 ± 3.71	Serum	mM Trolox equivalent	0.59 ± 0.20	27 (F)	0.75 ± 0.21	25(F)	<0.005	Erel [55]
					GCF Conc		0.09 ± 0.03		0.17 ± 0.04			
					GCF TAC/30s		0.40 ± 0.11		0.59 ± 0.10			
Su et al. [13]	USA	CS–CC	Scully and Langley-Evans [51]	52.27 (14.08)/45.39 (18.75) SEM	Saliva	mmol	0.71 ± 0.08	58 (25/33)	0.46 ± 0.04	234 (107/126)	0.0001	Miller et al. [58]
Kim et al. [14]	Korea	Interventional study	PPD ≥ 5 mm at least two sites per quadrant, BOP, radiographic LB ≥ 30%	50.0 ± 12.5/31.7 ± 8.0	Saliva	*μ*M	335.7 ± 36.6	7 (3/4)	282.7 ± 55.1	7 (3/4)		ImAnOx (TAS) Kit
Sulaiman and Shehadeh [15]	Syria	Interventional study	PPD ≥ 5 mm at least two sites per quadrant, BOP, radiographic LB ≥ 30%	41/34	Plasma	*µ*mol TEq	559 ± 53.2	30 (9/21)	625 ± 88.7	30 (9/21)	<0.001	Erel [55]
Dhotre et al. [17]	India	CC	Armitage [47]	52.7 ± 9.27/50.3 ± 9.39	Plasma	mmol/l	1.3 ± 0.15	100 (60/40)	2.32 ± 0.24	100 (60/40)	<0.001	Benzie and Strain [59]
					Saliva		0.46 ± 0.07		1.07 ± 0.03			
Dhotre et al. [18]	India	CC	Armitage [47]	52.7 ± 9.27/50.03 ± 9.39	Plasma	mmol/l	1.14 ± 0.13	50	2.32 ± 0.24	25	<0.001	Benzie and Strain [59]
Esen et al. [19]	Turkey	CS	AL ≥ 4 mm and PPD ≥ 5 mm.	42.85 ± 9.6/40.05 ± 9.8	Serum	millimoles trolox equivalent per/l	1.980 (1.835– 2.195)	20 (4/16)	2.510 (2.170– 2.725)	20 (4/16)	<0.001	Erel [55]
					GCF		0.0875 (0.059 to 0.140)		0.105 (0.070– 0.145)			
Konuganti et al. [20]	India	CC	PPD ≥ 5 mm at least 2 sites per quadrant, BOP	18–40 years	Blood	*µ*g/dl	37.13 ± 7.14	15	52.4 ± 9.71	15	<0.001	NBT method
Toker et al. [21]	Turkey	Interventional study	CAL > 30% of sites; PPD ≥ 5 mm	38.7 ± 5.9/38.0 ± 7.2	GCF	mmol TEq/l	0.12 ± 0.33	15 (7/8)	0.13 ± 0.25	10 (6/4)	>0.05	Erel [55]
Akpinar et al. [22]	Turkey	Interventional	Armitage [47]	37.7 ± 5.9/37.0 ± 7.4	GCF	mmol Trolox equiv./l	(0–0.1) 0.1	15 (7/8)	(0.1–0.1) 0.1	10 (5/5)		Erel [55]
Novaković et al. [23]	Serbia	PS	Bone loss >30%, at least one pocket; PPD >5 mm per quadrant with BOP	39.2 ± 11.5/35.2 ± 7.1	Saliva	*µ*mol/l	0.4 ± 0.24	21 (14/7)	0.59 ± 0.14	21 (14/7)	>0.05	ABTS colorimetric, Ransod kit
Sezer et al. [24]	Turkey	CS	–	45.50 ± 7.50/40.75 ± 10.26	Serum	mmol Trolox Eq/l	1.15 ± 0.36	20 (6/14)	1.30 ± 0.23	20 (6/14)		Erel [55]
Azizi et al. [25]	India	CC	Armitage [47]	37–50/39.64 ± 5.04	Plasma	*μ*mol/l	831.75 ± 78.15	134 (M)	925.2 ± 68.4	64 (M)	0.001	Benzie and Strain [59]
Baltacıoğlu et al. [26]	Turkey	CS and CC	Armitage [47]	32.55 ± 5.32/30.10 ± 4.06	Serum	mmol TEq	1.08 ± 0.0736	33 (16/17)	1.5 ± 0.0736	30 (16/14)	0.001	ABTS Erel [55]
					Saliva	mmol TEq	0.5 ± 0.11		0.71 ± 0.08		<0.05	Elisa kit
Bostanci [27]	Turkey	Interventional studies	Armitage [47]	38.80 ± 4.87/37.33 ± 5.67	GCF	*μ*mol trolox equivalent/l	0.09 (0.07–0.11)	15 (6/9)	0.07 (0.05–0.15)	15 (7/8)	>0.05	Erel [55]
					Serum		1.45 (1.23–2.10)		1.44 (1.28–1.68)			
Kose et al. [28]	Turkey	Comparative study	Armitage [47]	27–51 years	Saliva	mmol TEq/l	0.77 ± 0.14	22 (11/11)	0.92 ± 0.22	22 (12/10)	<0.05	Erel [55]
					Serum	mmol TEq/l	1.16 ± 0.29		1.34 ± 0.32			
Miricescu et al. [29]	Romania	CC	At least six sites with PD ≥ 4 mm; bone loss higher than 30% and gingival inflammation	51.26 ± 7.4/18.66 ± 2	Saliva	nmol/mg albumin	0.75 ± 0.16	25 (14/11)	1.24 ± 0.16	25 (20/5)	<0.05	ABTS colorimetric, Ransod kit
Thomas et al. [31]	India	Interventional	CAL ≥ 4 mm in at least 30% of sites	35–65	Serum	mmol/l	0.4972 ± 0.2250	25	1.2585 ± 0.3683	25	<0.001	–
Almerich-Silla et al [32]	Spain	CC	At least four zones with P*P* ≥ 5 mm and LA ≥ 2 mm	41–45/38–43	Saliva	mmol	1.09 ± 0.0905	33 (19/14)	0.9067 ± 0.119	37 (15/22)	<0.001	Elisa kit
Baser et al. [33]	Turkey	CC	Armitage [47]	39.9 ± 5.2/37.3 ± 5.4	Saliva	*μ*mol	0.33 ± 0.3	36 (11/25)	0.57 ± 0.3	16 (6/10)	<0.05	Commercially available calorimetry
					Plasma	*μ*mol	0.27 ± 0.14		0.44 ± 0.26			
Shankarram et al. [34]	India	CC	–	–	Saliva	mmol	1.03 ± 0.158	25	0.906 ± 0.119	25	<0.01	Elisa kit
Nguyen et al. [35]	Vietnam	CS	Armitage [47]	51.04 ± 12.25/51.17 ± 11.88	Saliva	mmol	0.07 ± 0.07	24 (12/12)	0.19 ± 0.14	24 (14/10)	<0.001	Antioxidant assay kit
Atabay et al. [36]	Turkey	CC	≥ 30% of sites with PPD ≥ 5 mm; with CAL ≥ 5 mm; ≥ 30% LB	42.47 ± 2.99/39.60 ± 5.84	GCF Concentration	*µ*M/ml	72.43 ± 6.79	15 (9/6)	351.60 ± 21.66	15 (6/9)	0.001	TAOC (ImAnOx-TAS)/TAC Kit
					GCF Total	*µ*M	69.70 ± 3.37		88.01 ± 4.61			
Bansel et al. [37]	India	Interventional	Armitage [47]	20–45	Plasma	*µ*mol/l	792.33 ± 124.33	40	1,076.1 ± 193.82	40	<0.001	Benzie and Strain [59]
Ahmadi-Motamayel et al. [38]	Iran	CC	Page and Eke [52]	30–50 (Both groups)	Saliva	mol/ml unit	0.16 ± 0.09	55 (28/27)	0.18 ± 0.1	56 (28/28)	0.11	Riviere and Papagiannoulis [60]
					Serum	mol/ml unit	0.36 ± 0.01		0.37 ± 0.05			
Punj et al. [39]	India	Comparative study	Armitage [48]	25–65 (Both groups)	Saliva	mmol/l	0.44 ± 0.14	20	0.48 ± 0.18	20	>0.05	Phosphomolybdenum method
					Serum	mmol/l	0.58 ± 0.13		0.94 ± 0.36		<0.001	
Narendra et al. [40]	India	CC	Armitage [47]	47.13 ± 7.00/36.56 ± 6.26	Serum	mmol/l	0.49 ± 0.075	46 (29/17)	1.23 ± 0.22	50 (33/17)	<0.001	Miller et al. [58]
					GCF	mmol/l	0.655 ± 0.16		1.36 ± 0.11			
Tripathi et al. [41]	India	CC	Armitage [47]	> 18 years	Saliva	mM Trolox equivalent	0.60	40	0.78	40	0.04	ELISA
					Serum		1.10		1.50		0.03	
Vincent et al. [42]	India	Comparative study	Armitage [48]	25–65 years	GCF	mmol/l	0.75 ± 0.24	20	1.2 ± 0.41	20	0.001	Erel [55]
Sánchez-Villamil et al. [43]	Colombia	CS and CC	Page and Eke [52]	45 ± 12/31 ± 10	Saliva	mmol	0.32 ± 0.21	87 (45/42)	0.15 ± 0.1	14 (6/8)	0.004	TAC assay kit (CS0790—Sigma- Aldrich)
Senouci et al. [44]	Algeria	CC	PPD ≥ 6 mm; with CAL ≥ 5 mm; Tooth loss due to periodontitis	24.06 ± 6.09/24.73 ± 1.38	Saliva	*μ*mol ascorbic acid eq/l	0.46 ± 0.34	29	1.45 ± 0.57	28	<0.0001	Kerboua et al. [61]
Thomas et al. [45]	India	RCT		30–60 (Both)	Serum	*µ*g/ml	0.5865 ± 0.1701	100	1.1028 ± 0.2600	100	<0.001	Phosphomolybdenum assay
Verghese et al. [46]	India	LS	Minimum 20 natural teeth; PPD at least 30% of sites; CAL >3 mm; bone loss	30–60 (Both)	Serum		0.56 ± 0.04	25	1.92 ± 0.10	25	<0.001	Phosphomolybdenum assay

SD, standard deviation; Cont, control; TEq, Trolox equivalent; ABTS, 2,2′-azinobis (3-ethylbenzothiazoline-6-sulfonic acid) FRAP, ferric reducing antioxidant power; TAC, total antioxidant capacity; PPD, periodontal pocket depth; BOP, bleeding on probing; LB, loss of bone; CAL, clinical attachment level; CC, case–control; PS, prospective study; CS, cross-sectional study; RCT, randomized controlled trial; LS, longitudinal study.

**Table 4:** The pre and posttreatment mean values of TAC in various biological fluid samples in patients with periodontitis in the included studies of quantitative synthesis.

Study name	Unit	Sample type	Periodontitis (pretreatment)		Periodontitis (posttreatment)		*p*-Value
			Mean ± SD or median (upper–lower value)	Sample size	Mean ± SD or median (upper–lower value)	Sample size	
Chapple et al. [8]	*μ*MTeq	Plasma	483 ± 111	35	489 ± 119	32	0.56
		GCF	632 ± 343		1,015 ± 549		0.001
Guentsch et al. [11]	*μ*mol/ml	Saliva	0.37 ± 0.24	15	0.44 ± 0.22	15	<0.05
Kim et al. [14]	*μ*M	Saliva	335.7 ± 36.6	7	326.8 ± 53.2	7	>0.05
Akpinar et al. [22]	mmol Trolox equiv./L	GCF	0.1 (0–0.1)	15	0.1 (0–0.1)	15	<0.05
Novaković et al. [23]	*µ*mol/l	Saliva	0.4 ± 0.24	21	0.66 ± 0.35	21	<0.01
Bostanci et al. [27]	*μ*mol Trolox equivalent/l	Serum	0.09 (0.07–0.11)	15	1.65 (1.55–2.62)	15	<0.05
		GCF	1.45 (1.23–2.10)		0.05 (0.04–0.09)		
Shirzaiy et al. [30]	*µ*mol/l	Saliva	0.655 ± 0.281	31	0.962 ± 0.287	31	<0.001
Thomas et al. [31]	mmol/l	Serum	0.497 ± 0.225	25	0.957 ± 0.188	25	≤0.001
Bansal [37]	*µ*mol/l	Plasma	792.33 ± 124.33	40	989.75 ± 96.80	40	<0.001
Verghese et al. [46]		Serum	0.56 ± 0.04	25	1.58 ± 0.05	25	<0.001

The treatment performed was non-surgical therapy (NST). SD, standard deviation.

## Data Availability

Data analyzed in this study were a reanalysis of existing data, which are openly available at locations cited in the reference section.

## Abbreviations

TOS: Total oxidative stress
TAC: Total anti-oxidant capacity
OS: Oxidative stress
NS: Nitrative stress
CI: Confidence interval
GCF: Gingival crevicular fluid
ROS: Reactive oxygen species
PI: Plaque index
GI: Gingival index
PPD: Periodontal probing depth
PD: Probing depth
BOP: Bleeding on probing
CAL: Clinical attachment level
LB: Loss of bone
FRAP: Ferric reducing antioxidant power
ABTS: 2,2′-azinobis-(3-ethylbenzothiazoline-6-sulfonate)
Eq: Equivalent
TEq: Trolox equivalent
CC: Case–control
CS: Cross-sectional
PS: Prospective study
LS: Longitudinal study
RCT: Randomized controlled trial.

## Additional Points

*Limitations*. The chronic exposure of the oral cavity to many environmental factors destabilizes the local redox homeostasis. It reduces the diagnostic usefulness of redox biomarkers in periodontal diseases. Furthermore, due to a lack of redox salivary/GCF biomarkers reference values for assessment, it is difficult to compare the values obtained from different studies. In addition, the resultant differences might be due to the different patient selection criteria in reported studies. The attributed factors such as age, gender, smoking, and nutrition status would yield distinct effects on the alteration of OS parameters; thus, it is essential to consider these factors when considering OS as a potential marker of periodontitis.
